# *catena*-Poly[triethyl­ammonium [[tetra-μ-acetato-κ^8^ *O*:*O*′-dicuprate(II)]-μ-acetato-κ^2^ *O*:*O*′] tetra­hydro­furan monosolvate]

**DOI:** 10.1107/S1600536812033405

**Published:** 2012-08-01

**Authors:** Bernhard E. C. Bugenhagen, Marc H. Prosenc

**Affiliations:** a Institute of Inorganic Chemistry, University of Hamburg, Hamburg, Germany; b Institute for Physical Chemistry, TU Kaiserslautern, Kaiserslautern, Germany

## Abstract

In the title compound, {[(C_2_H_5_)_3_NH][Cu_2_(CH_3_COO)_5_]·C_4_H_8_O}_*n*_, the two different Cu^II^ atoms are coordinated in a pseudo-square-pyramidal environment by five O atoms from the acetate ligands. Neighbouring pairs of Cu^II^ atoms are linked by four basally coordinating bridging acetate ligands as in the crystal structure of copper acetate monohydrate. The fifth, apically coordinating ligand links two of the dicopper tetra­acetate paddlewheel-units together, thus building a linear coordination polymer which extends along [10-1]. Each apical acetate ligand is linked by an N—H⋯O hydrogen bond to a triethyl­ammonium cation. Weak C—H⋯O hydrogen bonding interactions also occur.

## Related literature

For the crystal structure of dicoppertetra­acetate dihydrate, see: van Niekerk & Schoening (1953 ▶); de Meester *et al.* (1973 ▶). For copper-based coordination polymers, see: Furukawa *et al.* (2008 ▶). The title compound was obtained as a minor byproduct in the synthesis of a copper–salene compound, see: Kleij *et al.* (2005 ▶).
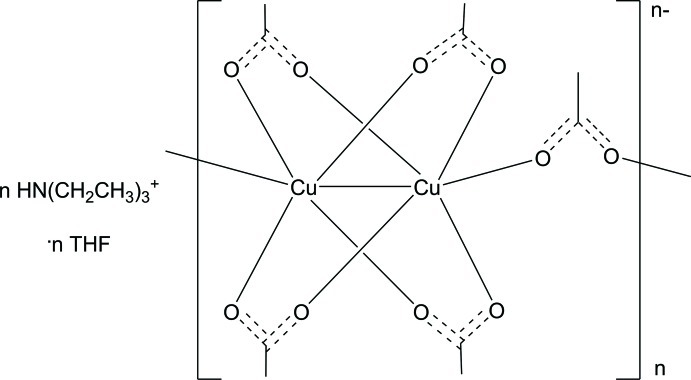


## Experimental

### 

#### Crystal data

(C_6_H_16_N)[Cu_2_(C_2_H_3_O_2_)_5_]·C_4_H_8_O
*M* *_r_* = 596.60
Monoclinic, 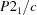

*a* = 12.1520 (2) Å
*b* = 12.2726 (2) Å
*c* = 18.7306 (3) Å
β = 112.956 (1)°
*V* = 2572.19 (7) Å^3^
*Z* = 4
Mo *K*α radiation
μ = 1.71 mm^−1^
*T* = 100 K
0.25 × 0.09 × 0.06 mm

#### Data collection

Bruker APEX II CCD area-detector diffractometer
Absorption correction: multi-scan (*SADABS*; Bruker, 2009 ▶) *T*_min_ = 0.681, *T*_max_ = 0.747
102874 measured reflections
4713 independent reflections
4047 reflections with *I* > 2σ(*I*)
*R*_int_ = 0.040

#### Refinement

*R*[*F* ^2^ > 2σ(*F* ^2^)] = 0.023
*wR*(*F* ^2^) = 0.062
*S* = 1.13
4713 reflections
319 parameters
H atoms treated by a mixture of independent and constrained refinement
Δρ_max_ = 0.32 e Å^−3^
Δρ_min_ = −0.36 e Å^−3^

### 

Data collection: *APEX2* (Bruker, 2009 ▶); cell refinement: *SAINT* (Bruker, 2009 ▶); data reduction: *SAINT*; program(s) used to solve structure: *SHELXS97* (Sheldrik, 2008 ▶); program(s) used to refine structure: *SHELXL97* (Sheldrik, 2008 ▶); molecular graphics: *OLEX2* (Dolomanov *et al.*, 2009 ▶); software used to prepare material for publication: *OLEX2*.

## Supplementary Material

Crystal structure: contains datablock(s) global, I. DOI: 10.1107/S1600536812033405/hp2044sup1.cif

Structure factors: contains datablock(s) I. DOI: 10.1107/S1600536812033405/hp2044Isup2.hkl

Supplementary material file. DOI: 10.1107/S1600536812033405/hp2044Isup3.mol

Additional supplementary materials:  crystallographic information; 3D view; checkCIF report

## Figures and Tables

**Table 1:** Selected bond lengths (Å)

Cu1—O1	1.9664 (14)
Cu1—O4	1.9717 (15)
Cu1—O3^i^	1.9738 (15)
Cu1—O2	1.9777 (15)
Cu1—O5	2.1216 (13)
Cu2—O7	1.9735 (14)
Cu2—O9	1.9804 (14)
Cu2—O8	1.9806 (14)
Cu2—O10	1.9839 (14)
Cu2—O6	2.1204 (13)

Symmetry code: (i) 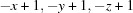.

**Table 2:** Hydrogen-bond geometry (Å, °)

*D*—H⋯*A*	*D*—H	H⋯*A*	*D*⋯*A*	*D*—H⋯*A*
N1—H1⋯O5	0.85 (2)	2.59 (2)	3.212 (2)	130.7 (19)
N1—H1⋯O6	0.85 (2)	1.89 (2)	2.737 (2)	173 (2)
C6—H6*C*⋯O1	0.98	2.42	3.3238 (18)	153
C13—H13*B*⋯O3^i^	0.99	2.51	3.2956 (19)	137
C15—H15*B*⋯O10	0.99	2.43	3.3041 (19)	147

Symmetry code: (i) 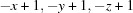.

**Fig. 1.**
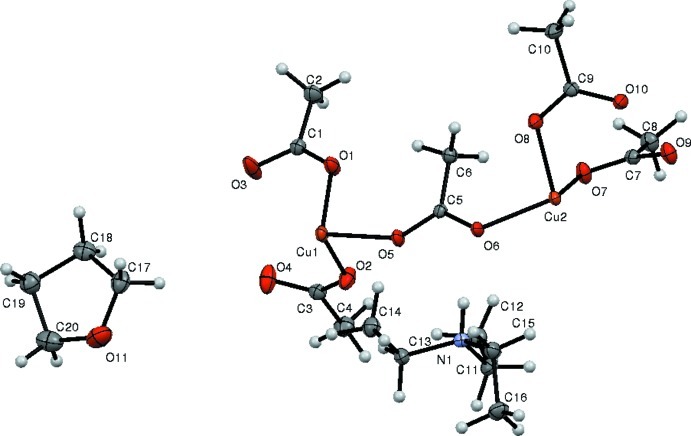
: Asymmertic unit of he title compound including the triethylammonium-cation and the co-crystallized THF-Molecule. Thermal ellipsoids are drawn at 50% probability.

**Fig. 2.**
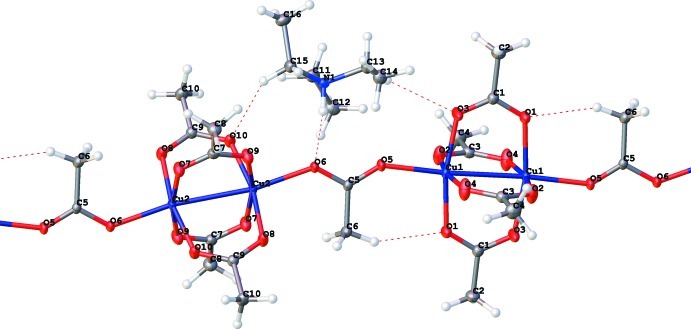
: Two units of dicoppertetraacetate linked by a bridging acetato-ligand. The three hydrogen-bonds between the triethylammonium-cation and the acetato-ligand as well as the short intramolecular contact between H6c and O1 are shown as red dashed lines. The THF-molecule has been omitted for clarity.

**Fig. 3.**
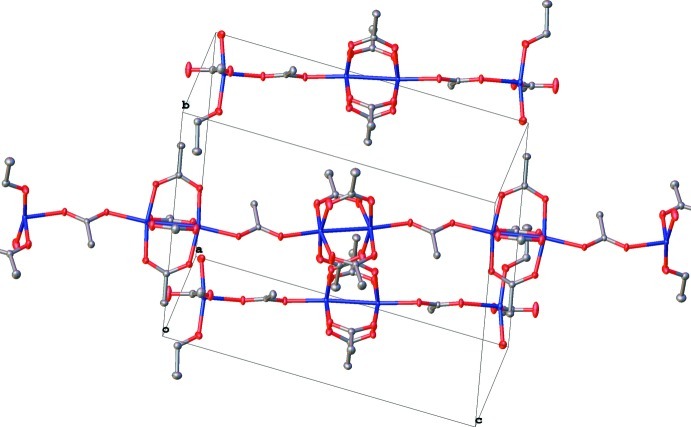
: Packing of the polymeric chains. The solvate-molecule, the cation and the hydrogen-atoms have been omitted for clarity. No direct inter-chain contact could be observed.

## supplementary crystallographic information

### Comment

The title compound, *catena*-poly[[triethylammonium [[tetra-µ-acetato-κ^8^*O*:*O*'-dicuprate(II)]-m-acetato-κ^2^*O*:*O*'] tetrahydrofuran monosolvate], was obtained as a minor byproduct in the synthesis of a copper-salene compound (Kleij *et al.*, 2005). The crystal structure consists of dicoppertetraacetate-paddlewheel-units with copper in a pseudo square-pyramidal coordination environment (Fig. 2). These monomeric units are interconnected by bridging, apically coordinating acetato-ligands, thus forming infinite zigzag chains along the [1 0 - 1] plane with an angle of about 165.4 ° with respect to the Cu—Cu bond-vectors (Fig. 3). Additionally, the monomers are skewed in respect to each other as indicated by the torsion-angle O1—Cu1—Cu2—O7 = -39.67 (7)° (see Fig.3, Table 1). One triethylammonium cation per dicoppertetraacetate monomer is present in the crystal structure. A hydrogen-bond between this cation and an oxygen-atom of the bridging acetato-ligand (O6) can be observed (*d*(N1—H1···O6) = 2.737 (2) Å, Table 2, Fig. 2). Two additional contacts are present between the coordination-chain and the alkyle-residues of the triethylammonium-cations (*d*(C15—H15B···O10) = 3.3041 (19) Å and d(C13—H13B···O3) = 3.2956 (19) Å, table 2). One short intra-chain contact is found between the methyl group of the bridging acetato-ligand (C6) and oxygen atom O1 of the monomeric unit with a distance of d(C6—H6C···O1) = 3.3238 (18) Å. However, no direct inter-chain contact could be observed. Furthermore, one molecule of tetrahydrofuran per dicoppertetraacetate-unit is present in the crystal structure. In spite of hydrogen-bonds being detected around this solvate-molecule, its orientation could be modeled unequivocally. The Cu—Cu distances in the monomers are d(Cu1—Cu1') = 2.6498 (4) Å and d(Cu2—Cu2') = 2.6542 (4) Å, respectively. This is significantly longer than the distance of 2.616 (1) Å found in dicoppertetraacetate-dihydrate (de Meester *et al.*, 1973). To the contrary, the apical Cu—O bonds in the title-compound are shorter (*d*(Cu1—O5) = 2.1216 (13) Å and d(Cu2—O6) = 2.1204 (13) Å) than the bond between copper and the aqua-ligand in dicoppertetraacetate-dihydrate (*d*(Cu—O(H2)) = 2.156 (4) Å). Finally it is to mention that, indicated by the slight differences in the Cu—Cu distances and the Cu—O bond-lengths and the Cu—Cu—O difference of the bond angles to the apically coordinating acetato-ligands mentioned in table 1, two neighbouring monomers exhibit a minor asymmetry. The aforementioned skewing and the position of the triethylammonoium-cation, especially the N—H···O hydrogen bond, are further indicators of this.

### Refinement

C-bound hydrogen atoms were placed in calculated positions with C—H distance of 0.99 - 1.00 Å and refined as riding with *U*_iso_(H) = *xU*_eq_(C), where *x* = 1.5 for methyl and *x* = 1.2 for all other H-atoms. H atoms on N atoms were located in a difference Fourier map and refined isotropically.

### Crystal data

**Table:**

(C_6_H_16_N)[Cu_2_(C_2_H_3_O_2_)_5_]·C_4_H_8_O	*F*(000) = 1248
*M**_r_* = 596.60	*D*_x_ = 1.541 Mg m^−^^3^
Monoclinic, *P*2_1_/*c*	Mo *K*α radiation, λ = 0.71073 Å
Hall symbol: -P 2ybc	Cell parameters from 9902 reflections
*a* = 12.1520 (2) Å	θ = 2.4–34.0°
*b* = 12.2726 (2) Å	µ = 1.71 mm^−^^1^
*c* = 18.7306 (3) Å	*T* = 100 K
β = 112.956 (1)°	Block, clear dark blue
*V* = 2572.19 (7) Å^3^	0.25 × 0.09 × 0.06 mm
*Z* = 4	

### Data collection

**Table:**

Bruker APEX II CCD area-detector diffractometer	4713 independent reflections
Radiation source: micro-focus	4047 reflections with *I* > 2σ(*I*)
Multi-layer optics monochromator	*R*_int_ = 0.040
Detector resolution: 8 pixels mm^-1^	θ_max_ = 25.4°, θ_min_ = 1.8°
ω and φ scans	*h* = −14→14
Absorption correction: multi-scan (*SADABS*; Bruker, 2009)	*k* = −14→14
*T*_min_ = 0.681, *T*_max_ = 0.747	*l* = −22→22
102874 measured reflections	

### Refinement

**Table:**

Refinement on *F*^2^	Primary atom site location: structure-invariant direct methods
Least-squares matrix: full	Secondary atom site location: difference Fourier map
*R*[*F*^2^ > 2σ(*F*^2^)] = 0.023	Hydrogen site location: inferred from neighbouring sites
*wR*(*F*^2^) = 0.062	H atoms treated by a mixture of independent and constrained refinement
*S* = 1.13	*w* = 1/[σ^2^(*F*_o_^2^) + (0.0203*P*)^2^ + 2.3331*P*] where *P* = (*F*_o_^2^ + 2*F*_c_^2^)/3
4713 reflections	(Δ/σ)_max_ = 0.004
319 parameters	Δρ_max_ = 0.32 e Å^−^^3^
0 restraints	Δρ_min_ = −0.36 e Å^−^^3^

### Special details

**Table:**

Experimental. Absorption correction: SADABS-2008/1 (Bruker, 2009) was used for absorption correction. wR2(int) was 0.1027 before and 0.0524 after correction. The Ratio of minimum to maximum transmission is 0.9107. The λ/2 correction factor is 0.0000.
Geometry. All e.s.d.'s (except the e.s.d. in the dihedral angle between two l.s. planes) are estimated using the full covariance matrix. The cell e.s.d.'s are taken into account individually in the estimation of e.s.d.'s in distances, angles and torsion angles; correlations between e.s.d.'s in cell parameters are only used when they are defined by crystal symmetry. An approximate (isotropic) treatment of cell e.s.d.'s is used for estimating e.s.d.'s involving l.s. planes.
Refinement. Refinement of *F*^2^ against ALL reflections. The weighted *R*-factor *wR* and goodness of fit *S* are based on *F*^2^, conventional *R*-factors *R* are based on *F*, with *F* set to zero for negative *F*^2^. The threshold expression of *F*^2^ > σ(*F*^2^) is used only for calculating *R*-factors(gt) *etc*. and is not relevant to the choice of reflections for refinement. *R*-factors based on *F*^2^ are statistically about twice as large as those based on *F*, and *R*- factors based on ALL data will be even larger.

### Fractional atomic coordinates and isotropic or equivalent isotropic displacement parameters (Å^2^)

**Table:**

	*x*	*y*	*z*	*U*_iso_*/*U*_eq_	
Cu1	0.58451 (2)	0.505128 (19)	0.570813 (12)	0.01381 (7)	
Cu2	0.937948 (19)	0.502200 (18)	0.923936 (12)	0.01216 (7)	
O1	0.45990 (12)	0.43975 (12)	0.59966 (8)	0.0217 (3)	
O2	0.51683 (14)	0.65182 (12)	0.57030 (8)	0.0275 (4)	
O3	0.31686 (13)	0.43082 (15)	0.48086 (8)	0.0336 (4)	
O4	0.62799 (13)	0.35832 (13)	0.54785 (9)	0.0295 (4)	
O5	0.72384 (12)	0.52443 (11)	0.68184 (7)	0.0169 (3)	
O6	0.85592 (12)	0.52508 (11)	0.80224 (7)	0.0154 (3)	
O7	0.80423 (12)	0.54543 (12)	0.95269 (8)	0.0211 (3)	
O8	0.90257 (13)	0.34674 (11)	0.93393 (8)	0.0203 (3)	
O9	1.09120 (12)	0.45715 (12)	0.91883 (8)	0.0211 (3)	
O10	0.99288 (13)	0.65590 (11)	0.93776 (8)	0.0203 (3)	
C1	0.35629 (17)	0.41637 (16)	0.55266 (11)	0.0170 (4)	
C2	0.27189 (19)	0.36747 (18)	0.58551 (12)	0.0231 (5)	
H2A	0.3171	0.3447	0.6392	0.035*	
H2B	0.2317	0.3041	0.5544	0.035*	
H2C	0.2121	0.4219	0.5842	0.035*	
C3	0.42955 (17)	0.69004 (16)	0.51461 (11)	0.0177 (4)	
C4	0.3902 (2)	0.80429 (18)	0.52395 (13)	0.0259 (5)	
H4A	0.3487	0.8030	0.5595	0.039*	
H4B	0.3360	0.8320	0.4734	0.039*	
H4C	0.4603	0.8518	0.5450	0.039*	
C5	0.75337 (16)	0.49915 (14)	0.75148 (10)	0.0123 (4)	
C6	0.66926 (17)	0.43909 (17)	0.77852 (11)	0.0179 (4)	
H6A	0.6513	0.4842	0.8158	0.027*	
H6B	0.7064	0.3708	0.8034	0.027*	
H6C	0.5951	0.4230	0.7340	0.027*	
C7	0.81493 (17)	0.55813 (16)	1.02184 (11)	0.0158 (4)	
C8	0.70571 (18)	0.59690 (18)	1.03438 (12)	0.0210 (4)	
H8A	0.6998	0.6764	1.0293	0.032*	
H8B	0.7125	0.5759	1.0864	0.032*	
H8C	0.6340	0.5635	0.9956	0.032*	
C9	0.94290 (17)	0.30002 (16)	0.99907 (11)	0.0150 (4)	
C10	0.91147 (18)	0.18182 (16)	1.00283 (12)	0.0199 (4)	
H10A	0.8507	0.1762	1.0250	0.030*	
H10B	0.9832	0.1416	1.0355	0.030*	
H10C	0.8801	0.1508	0.9505	0.030*	
N1	0.95203 (15)	0.68314 (14)	0.74095 (9)	0.0157 (3)	
C11	0.89976 (19)	0.78890 (17)	0.75415 (12)	0.0216 (4)	
H11A	0.9146	0.8465	0.7220	0.026*	
H11B	0.9409	0.8104	0.8092	0.026*	
C12	0.76660 (19)	0.78157 (18)	0.73454 (13)	0.0250 (5)	
H12A	0.7520	0.7316	0.7708	0.037*	
H12B	0.7260	0.7543	0.6815	0.037*	
H12C	0.7357	0.8540	0.7387	0.037*	
C13	0.92652 (18)	0.66676 (16)	0.65642 (11)	0.0178 (4)	
H13A	0.9663	0.7252	0.6388	0.021*	
H13B	0.8394	0.6730	0.6262	0.021*	
C14	0.96895 (19)	0.55746 (17)	0.64007 (12)	0.0230 (5)	
H14A	0.9390	0.4997	0.6639	0.034*	
H14B	1.0565	0.5560	0.6618	0.034*	
H14C	0.9388	0.5458	0.5839	0.034*	
C15	1.08264 (18)	0.67378 (18)	0.79283 (12)	0.0208 (4)	
H15A	1.1132	0.6029	0.7832	0.025*	
H15B	1.0912	0.6749	0.8476	0.025*	
C16	1.15768 (19)	0.76417 (18)	0.78030 (13)	0.0259 (5)	
H16A	1.2415	0.7536	0.8151	0.039*	
H16B	1.1296	0.8345	0.7914	0.039*	
H16C	1.1506	0.7630	0.7264	0.039*	
O11	0.68670 (15)	0.43535 (14)	0.26275 (10)	0.0376 (4)	
C17	0.6524 (2)	0.36448 (19)	0.31112 (13)	0.0279 (5)	
H17A	0.6561	0.4038	0.3582	0.034*	
H17B	0.7072	0.3013	0.3274	0.034*	
C18	0.52537 (19)	0.32611 (18)	0.26465 (13)	0.0255 (5)	
H18A	0.4663	0.3716	0.2754	0.031*	
H18B	0.5146	0.2490	0.2759	0.031*	
C19	0.5144 (2)	0.34046 (19)	0.18108 (13)	0.0293 (5)	
H19A	0.5431	0.2750	0.1626	0.035*	
H19B	0.4308	0.3555	0.1458	0.035*	
C20	0.5939 (2)	0.4374 (2)	0.18684 (15)	0.0346 (6)	
H20A	0.6284	0.4327	0.1470	0.041*	
H20B	0.5476	0.5059	0.1788	0.041*	
H1	0.918 (2)	0.632 (2)	0.7560 (13)	0.021 (6)*	

### Atomic displacement parameters (Å^2^)

**Table:**

	*U*^11^	*U*^22^	*U*^33^	*U*^12^	*U*^13^	*U*^23^
Cu1	0.01246 (12)	0.01771 (13)	0.00901 (12)	−0.00095 (9)	0.00175 (9)	0.00001 (9)
Cu2	0.01315 (12)	0.01314 (12)	0.00850 (12)	−0.00060 (9)	0.00238 (9)	0.00073 (9)
O1	0.0174 (7)	0.0319 (8)	0.0128 (7)	−0.0052 (6)	0.0027 (6)	0.0026 (6)
O2	0.0324 (9)	0.0187 (8)	0.0196 (8)	0.0037 (7)	−0.0027 (7)	−0.0015 (6)
O3	0.0194 (8)	0.0648 (12)	0.0126 (7)	−0.0166 (8)	0.0020 (6)	0.0037 (8)
O4	0.0259 (8)	0.0317 (9)	0.0204 (8)	0.0122 (7)	−0.0022 (6)	−0.0061 (7)
O5	0.0154 (7)	0.0231 (7)	0.0085 (6)	−0.0025 (6)	0.0007 (5)	0.0013 (5)
O6	0.0148 (7)	0.0179 (7)	0.0096 (6)	−0.0037 (5)	0.0007 (5)	0.0013 (5)
O7	0.0167 (7)	0.0308 (8)	0.0147 (7)	0.0024 (6)	0.0050 (6)	−0.0011 (6)
O8	0.0247 (7)	0.0162 (7)	0.0137 (7)	−0.0020 (6)	0.0007 (6)	0.0017 (6)
O9	0.0178 (7)	0.0307 (8)	0.0139 (7)	0.0050 (6)	0.0051 (6)	0.0018 (6)
O10	0.0275 (8)	0.0158 (7)	0.0141 (7)	−0.0045 (6)	0.0043 (6)	0.0002 (6)
C1	0.0183 (10)	0.0164 (10)	0.0170 (10)	0.0010 (8)	0.0076 (8)	−0.0008 (8)
C2	0.0226 (11)	0.0275 (12)	0.0210 (11)	−0.0034 (9)	0.0103 (9)	0.0004 (9)
C3	0.0181 (10)	0.0191 (10)	0.0185 (10)	−0.0008 (8)	0.0101 (8)	0.0024 (8)
C4	0.0281 (12)	0.0214 (11)	0.0282 (12)	0.0028 (9)	0.0109 (10)	−0.0006 (9)
C5	0.0139 (9)	0.0083 (9)	0.0140 (9)	0.0010 (7)	0.0046 (8)	−0.0016 (7)
C6	0.0157 (10)	0.0205 (10)	0.0143 (9)	−0.0019 (8)	0.0024 (8)	0.0008 (8)
C7	0.0188 (10)	0.0113 (9)	0.0176 (10)	−0.0024 (8)	0.0074 (8)	0.0002 (8)
C8	0.0193 (10)	0.0240 (11)	0.0212 (10)	0.0010 (8)	0.0094 (9)	0.0000 (9)
C9	0.0136 (9)	0.0157 (10)	0.0156 (10)	0.0020 (8)	0.0057 (8)	0.0009 (8)
C10	0.0218 (10)	0.0161 (10)	0.0189 (10)	−0.0013 (8)	0.0047 (8)	0.0015 (8)
N1	0.0184 (8)	0.0137 (8)	0.0157 (8)	−0.0030 (7)	0.0073 (7)	−0.0003 (7)
C11	0.0292 (11)	0.0154 (10)	0.0214 (11)	−0.0020 (8)	0.0111 (9)	−0.0018 (8)
C12	0.0281 (12)	0.0214 (11)	0.0290 (12)	0.0048 (9)	0.0149 (10)	0.0011 (9)
C13	0.0208 (10)	0.0182 (10)	0.0132 (9)	−0.0015 (8)	0.0053 (8)	0.0002 (8)
C14	0.0260 (11)	0.0220 (11)	0.0224 (11)	−0.0005 (9)	0.0110 (9)	−0.0035 (9)
C15	0.0193 (10)	0.0253 (11)	0.0159 (10)	−0.0025 (9)	0.0047 (8)	0.0016 (9)
C16	0.0238 (11)	0.0251 (12)	0.0305 (12)	−0.0058 (9)	0.0124 (10)	−0.0032 (10)
O11	0.0344 (9)	0.0347 (10)	0.0437 (10)	−0.0099 (8)	0.0151 (8)	−0.0029 (8)
C17	0.0300 (12)	0.0254 (12)	0.0281 (12)	0.0035 (9)	0.0110 (10)	−0.0050 (10)
C18	0.0254 (12)	0.0222 (11)	0.0295 (12)	0.0057 (9)	0.0114 (10)	0.0024 (9)
C19	0.0284 (12)	0.0298 (12)	0.0281 (12)	0.0012 (10)	0.0091 (10)	0.0000 (10)
C20	0.0373 (14)	0.0307 (13)	0.0414 (14)	0.0035 (11)	0.0216 (12)	0.0080 (11)

### Geometric parameters (Å, º)

**Table:**

Cu1—O1	1.9664 (14)	C9—O10^ii^	1.258 (2)
Cu1—O4	1.9717 (15)	C9—C10	1.509 (3)
Cu1—O3^i^	1.9738 (15)	C10—H10A	0.9800
Cu1—O2	1.9777 (15)	C10—H10B	0.9800
Cu1—O5	2.1216 (13)	C10—H10C	0.9800
Cu1—Cu1^i^	2.6498 (4)	N1—C13	1.504 (2)
Cu2—O7	1.9735 (14)	N1—C11	1.507 (3)
Cu2—O9	1.9804 (14)	N1—C15	1.508 (3)
Cu2—O8	1.9806 (14)	N1—H1	0.85 (2)
Cu2—O10	1.9839 (14)	C11—C12	1.516 (3)
Cu2—O6	2.1204 (13)	C11—H11A	0.9900
Cu2—Cu2^ii^	2.6542 (4)	C11—H11B	0.9900
O1—C1	1.256 (2)	C12—H12A	0.9800
O2—C3	1.253 (2)	C12—H12B	0.9800
O3—C1	1.252 (2)	C12—H12C	0.9800
O3—Cu1^i^	1.9739 (15)	C13—C14	1.511 (3)
O4—C3^i^	1.254 (2)	C13—H13A	0.9900
O5—C5	1.250 (2)	C13—H13B	0.9900
O6—C5	1.278 (2)	C14—H14A	0.9800
O7—C7	1.260 (2)	C14—H14B	0.9800
O8—C9	1.261 (2)	C14—H14C	0.9800
O9—C7^ii^	1.258 (2)	C15—C16	1.511 (3)
O10—C9^ii^	1.258 (2)	C15—H15A	0.9900
C1—C2	1.511 (3)	C15—H15B	0.9900
C2—H2A	0.9800	C16—H16A	0.9800
C2—H2B	0.9800	C16—H16B	0.9800
C2—H2C	0.9800	C16—H16C	0.9800
C3—O4^i^	1.254 (2)	O11—C20	1.429 (3)
C3—C4	1.513 (3)	O11—C17	1.430 (3)
C4—H4A	0.9800	C17—C18	1.521 (3)
C4—H4B	0.9800	C17—H17A	0.9900
C4—H4C	0.9800	C17—H17B	0.9900
C5—C6	1.499 (3)	C18—C19	1.529 (3)
C6—H6A	0.9800	C18—H18A	0.9900
C6—H6B	0.9800	C18—H18B	0.9900
C6—H6C	0.9800	C19—C20	1.510 (3)
C7—O9^ii^	1.258 (2)	C19—H19A	0.9900
C7—C8	1.512 (3)	C19—H19B	0.9900
C8—H8A	0.9800	C20—H20A	0.9900
C8—H8B	0.9800	C20—H20B	0.9900
C8—H8C	0.9800		
			
O1—Cu1—O4	89.30 (7)	O10^ii^—C9—O8	125.38 (18)
O1—Cu1—O3^i^	167.73 (6)	O10^ii^—C9—C10	116.43 (17)
O4—Cu1—O3^i^	89.50 (8)	O8—C9—C10	118.18 (17)
O1—Cu1—O2	90.54 (7)	C9—C10—H10A	109.5
O4—Cu1—O2	167.71 (6)	C9—C10—H10B	109.5
O3^i^—Cu1—O2	88.05 (8)	H10A—C10—H10B	109.5
O1—Cu1—O5	100.48 (5)	C9—C10—H10C	109.5
O4—Cu1—O5	97.85 (6)	H10A—C10—H10C	109.5
O3^i^—Cu1—O5	91.78 (6)	H10B—C10—H10C	109.5
O2—Cu1—O5	94.27 (6)	C13—N1—C11	111.15 (15)
O1—Cu1—Cu1^i^	82.90 (4)	C13—N1—C15	113.66 (15)
O4—Cu1—Cu1^i^	84.68 (4)	C11—N1—C15	111.40 (16)
O3^i^—Cu1—Cu1^i^	84.83 (4)	C13—N1—H1	108.7 (15)
O2—Cu1—Cu1^i^	83.10 (4)	C11—N1—H1	106.9 (16)
O5—Cu1—Cu1^i^	175.77 (4)	C15—N1—H1	104.5 (15)
O7—Cu2—O9	167.99 (6)	N1—C11—C12	112.80 (17)
O7—Cu2—O8	90.06 (6)	N1—C11—H11A	109.0
O9—Cu2—O8	88.77 (6)	C12—C11—H11A	109.0
O7—Cu2—O10	88.84 (6)	N1—C11—H11B	109.0
O9—Cu2—O10	89.82 (6)	C12—C11—H11B	109.0
O8—Cu2—O10	167.95 (6)	H11A—C11—H11B	107.8
O7—Cu2—O6	99.55 (5)	C11—C12—H12A	109.5
O9—Cu2—O6	92.39 (5)	C11—C12—H12B	109.5
O8—Cu2—O6	101.58 (5)	H12A—C12—H12B	109.5
O10—Cu2—O6	90.44 (5)	C11—C12—H12C	109.5
O7—Cu2—Cu2^ii^	84.03 (4)	H12A—C12—H12C	109.5
O9—Cu2—Cu2^ii^	83.96 (4)	H12B—C12—H12C	109.5
O8—Cu2—Cu2^ii^	86.12 (4)	N1—C13—C14	112.69 (16)
O10—Cu2—Cu2^ii^	81.83 (4)	N1—C13—H13A	109.1
O6—Cu2—Cu2^ii^	171.44 (4)	C14—C13—H13A	109.1
C1—O1—Cu1	124.71 (13)	N1—C13—H13B	109.1
C3—O2—Cu1	124.07 (13)	C14—C13—H13B	109.1
C1—O3—Cu1^i^	122.14 (13)	H13A—C13—H13B	107.8
C3^i^—O4—Cu1	122.47 (13)	C13—C14—H14A	109.5
C5—O5—Cu1	142.18 (12)	C13—C14—H14B	109.5
C5—O6—Cu2	132.82 (12)	H14A—C14—H14B	109.5
C7—O7—Cu2	123.23 (13)	C13—C14—H14C	109.5
C9—O8—Cu2	120.83 (13)	H14A—C14—H14C	109.5
C7^ii^—O9—Cu2	123.01 (13)	H14B—C14—H14C	109.5
C9^ii^—O10—Cu2	125.84 (13)	N1—C15—C16	113.03 (17)
O3—C1—O1	125.42 (18)	N1—C15—H15A	109.0
O3—C1—C2	117.23 (18)	C16—C15—H15A	109.0
O1—C1—C2	117.35 (17)	N1—C15—H15B	109.0
C1—C2—H2A	109.5	C16—C15—H15B	109.0
C1—C2—H2B	109.5	H15A—C15—H15B	107.8
H2A—C2—H2B	109.5	C15—C16—H16A	109.5
C1—C2—H2C	109.5	C15—C16—H16B	109.5
H2A—C2—H2C	109.5	H16A—C16—H16B	109.5
H2B—C2—H2C	109.5	C15—C16—H16C	109.5
O2—C3—O4^i^	125.63 (19)	H16A—C16—H16C	109.5
O2—C3—C4	116.98 (18)	H16B—C16—H16C	109.5
O4^i^—C3—C4	117.39 (18)	C20—O11—C17	109.24 (17)
C3—C4—H4A	109.5	O11—C17—C18	107.89 (18)
C3—C4—H4B	109.5	O11—C17—H17A	110.1
H4A—C4—H4B	109.5	C18—C17—H17A	110.1
C3—C4—H4C	109.5	O11—C17—H17B	110.1
H4A—C4—H4C	109.5	C18—C17—H17B	110.1
H4B—C4—H4C	109.5	H17A—C17—H17B	108.4
O5—C5—O6	120.95 (17)	C17—C18—C19	102.34 (18)
O5—C5—C6	121.24 (16)	C17—C18—H18A	111.3
O6—C5—C6	117.81 (16)	C19—C18—H18A	111.3
C5—C6—H6A	109.5	C17—C18—H18B	111.3
C5—C6—H6B	109.5	C19—C18—H18B	111.3
H6A—C6—H6B	109.5	H18A—C18—H18B	109.2
C5—C6—H6C	109.5	C20—C19—C18	102.71 (19)
H6A—C6—H6C	109.5	C20—C19—H19A	111.2
H6B—C6—H6C	109.5	C18—C19—H19A	111.2
O9^ii^—C7—O7	125.74 (18)	C20—C19—H19B	111.2
O9^ii^—C7—C8	117.27 (17)	C18—C19—H19B	111.2
O7—C7—C8	116.98 (17)	H19A—C19—H19B	109.1
C7—C8—H8A	109.5	O11—C20—C19	106.94 (19)
C7—C8—H8B	109.5	O11—C20—H20A	110.3
H8A—C8—H8B	109.5	C19—C20—H20A	110.3
C7—C8—H8C	109.5	O11—C20—H20B	110.3
H8A—C8—H8C	109.5	C19—C20—H20B	110.3
H8B—C8—H8C	109.5	H20A—C20—H20B	108.6
			
O4—Cu1—O1—C1	−84.84 (17)	O8—Cu2—O9—C7^ii^	−85.90 (16)
O3^i^—Cu1—O1—C1	−0.4 (4)	O10—Cu2—O9—C7^ii^	82.13 (16)
O2—Cu1—O1—C1	82.87 (17)	O6—Cu2—O9—C7^ii^	172.55 (16)
O5—Cu1—O1—C1	177.31 (16)	Cu2^ii^—Cu2—O9—C7^ii^	0.33 (15)
Cu1^i^—Cu1—O1—C1	−0.12 (16)	O7—Cu2—O10—C9^ii^	83.93 (16)
O1—Cu1—O2—C3	−81.93 (17)	O9—Cu2—O10—C9^ii^	−84.13 (16)
O4—Cu1—O2—C3	7.2 (4)	O8—Cu2—O10—C9^ii^	−0.9 (4)
O3^i^—Cu1—O2—C3	85.87 (17)	O6—Cu2—O10—C9^ii^	−176.52 (16)
O5—Cu1—O2—C3	177.52 (17)	Cu2^ii^—Cu2—O10—C9^ii^	−0.21 (15)
Cu1^i^—Cu1—O2—C3	0.85 (16)	Cu1^i^—O3—C1—O1	0.0 (3)
O1—Cu1—O4—C3^i^	80.50 (17)	Cu1^i^—O3—C1—C2	179.53 (14)
O3^i^—Cu1—O4—C3^i^	−87.28 (17)	Cu1—O1—C1—O3	0.1 (3)
O2—Cu1—O4—C3^i^	−8.8 (4)	Cu1—O1—C1—C2	−179.43 (14)
O5—Cu1—O4—C3^i^	−179.01 (16)	Cu1—O2—C3—O4^i^	0.8 (3)
Cu1^i^—Cu1—O4—C3^i^	−2.43 (16)	Cu1—O2—C3—C4	−179.51 (14)
O1—Cu1—O5—C5	10.1 (2)	Cu1—O5—C5—O6	175.75 (13)
O4—Cu1—O5—C5	−80.6 (2)	Cu1—O5—C5—C6	−5.2 (3)
O3^i^—Cu1—O5—C5	−170.3 (2)	Cu2—O6—C5—O5	178.19 (12)
O2—Cu1—O5—C5	101.5 (2)	Cu2—O6—C5—C6	−0.9 (3)
Cu1^i^—Cu1—O5—C5	153.0 (5)	Cu2—O7—C7—O9^ii^	−1.9 (3)
O7—Cu2—O6—C5	−45.10 (17)	Cu2—O7—C7—C8	177.02 (13)
O9—Cu2—O6—C5	136.17 (16)	Cu2—O8—C9—O10^ii^	0.0 (3)
O8—Cu2—O6—C5	46.94 (17)	Cu2—O8—C9—C10	179.31 (13)
O10—Cu2—O6—C5	−133.99 (16)	C13—N1—C11—C12	78.9 (2)
Cu2^ii^—Cu2—O6—C5	−159.3 (2)	C15—N1—C11—C12	−153.20 (17)
O9—Cu2—O7—C7	3.0 (4)	C11—N1—C13—C14	−175.42 (17)
O8—Cu2—O7—C7	87.31 (16)	C15—N1—C13—C14	57.9 (2)
O10—Cu2—O7—C7	−80.69 (16)	C13—N1—C15—C16	66.7 (2)
O6—Cu2—O7—C7	−170.94 (15)	C11—N1—C15—C16	−59.8 (2)
Cu2^ii^—Cu2—O7—C7	1.21 (15)	C20—O11—C17—C18	−4.7 (2)
O7—Cu2—O8—C9	−83.88 (15)	O11—C17—C18—C19	23.3 (2)
O9—Cu2—O8—C9	84.17 (15)	C17—C18—C19—C20	−31.8 (2)
O10—Cu2—O8—C9	0.8 (4)	C17—O11—C20—C19	−16.4 (2)
O6—Cu2—O8—C9	176.36 (14)	C18—C19—C20—O11	30.4 (2)
Cu2^ii^—Cu2—O8—C9	0.14 (14)	O1—Cu1—Cu2—O7	−39.67 (7)
O7—Cu2—O9—C7^ii^	−1.4 (4)		

Symmetry codes: (i) −*x*+1, −*y*+1, −*z*+1; (ii) −*x*+2, −*y*+1, −*z*+2.

### Hydrogen-bond geometry (Å, º)

**Table:**

*D*—H···*A*	*D*—H	H···*A*	*D*···*A*	*D*—H···*A*
N1—H1···O5	0.85 (2)	2.59 (2)	3.212 (2)	130.7 (19)
N1—H1···O6	0.85 (2)	1.89 (2)	2.737 (2)	173 (2)
C6—H6*C*···O1	0.98	2.42	3.3238 (18)	153
C13—H13*B*···O3^i^	0.99	2.51	3.2956 (19)	137
C15—H15*B*···O10	0.99	2.43	3.3041 (19)	147

Symmetry code: (i) −*x*+1, −*y*+1, −*z*+1.
